# Outcomes by Race and Ethnicity Following a Medicare Bundled Payment Program for Joint Replacement

**DOI:** 10.1001/jamanetworkopen.2024.33962

**Published:** 2024-09-17

**Authors:** Narae Kim, Mireille Jacobson

**Affiliations:** 1 Leonard Davis School of Gerontology, University of Southern California, Los Angeles; 2 USC Leonard D. Schaeffer Center for Health Policy & Economics, Los Angeles, California

## Abstract

**Importance:**

The Comprehensive Care for Joint Replacement (CJR) model, a traditional Medicare bundled payment program for lower-extremity joint replacement, is associated with care for patients outside traditional Medicare. Whether CJR model outcomes have differed by patient race or ethnicity outside of traditional Medicare is unclear.

**Objective:**

To evaluate outcomes associated with the CJR model among Hispanic patients not enrolled in traditional Medicare.

**Design, Setting, and Participants:**

This cohort study used hospitalization data from California’s Patient Discharge Dataset for all patients who underwent lower-extremity joint replacement in California between January 1, 2014, and December 31, 2017. In California, 3 metropolitan statistical areas (MSAs) were randomly selected to participate in CJR in April 2016. Hospitals not participating in other Medicare Alternative Payment Models were included in the treated group if they were in these 3 MSAs and in the control group if they were in the remaining 23 MSAs. The data analysis was performed between October 1 and December 31, 2023.

**Exposure:**

Comprehensive Care for Joint Replacement program implementation.

**Main Outcomes and Measures:**

The main outcomes were hospital length of stay and home discharge rates by race and ethnicity. Home discharge status included self-care, the use of home health services, and hospice care at home. Event study, difference-in-differences, and triple differences models were used to estimate differential changes in health care service use by race and ethnicity for patients in the treated MSAs compared with the control MSAs before vs after CJR implementation.

**Results:**

Of 309 834 hospitalizations (patient mean [SD] age, 68.3 [11.3] years; 60.6% women; 14.8% Hispanic; 72.4% non-Hispanic White), 48.0% were in treated MSAs and 52.0% in control MSAs. The CJR program was associated with an increase in home discharge rates for patients without traditional Medicare coverage; however, the increase differed by patient race and ethnicity. The increase was 0.05 (95% CI, 0.02-0.08) percentage points higher for Hispanic patients with Medicare Advantage and 0.03 (95% CI, 0.01-0.04) percentage points higher for Hispanic patients without Medicare compared with their non-Hispanic White counterparts.

**Conclusions and Relevance:**

This cohort study shows that CJR program outcomes differed by race and ethnicity for patients covered outside traditional Medicare, with home discharge rates increasing more for Hispanic compared with non-Hispanic White patients. These findings suggest the importance of considering differential outcomes of Medicare payment policies for racial and ethnic minority patient populations beyond the initially targeted groups.

## Key Points

**Question:**

What outcomes were associated with race and ethnicity following implementation of the Comprehensive Care for Joint Replacement (CJR) program, a traditional Medicare bundled payment model?

**Findings:**

In this cohort study of 309 834 hospitalizations for lower-extremity joint replacement in California from 2014 to 2017, the CJR model was associated with a larger increase in home discharge rates for Hispanic patients with Medicare Advantage vs non-Medicare coverage compared with their non-Hispanic White counterparts.

**Meaning:**

These findings suggest that the CJR model may increase home discharge rates among Hispanic patients outside of traditional Medicare and thereby reduce discrepancy in postacute care.

## Teaser

This cohort study evaluates outcomes for Hispanic and White patients associated with Medicare’s Comprehensive Care for Joint Replacement bundled payment program in California.

## Introduction

Lower-extremity joint replacement (LEJR), which encompasses procedures such as total hip arthroplasty and total knee arthroplasty, is one of the most prevalent surgical interventions among older adults in the US.^1^ Approximately 1 million total hip and knee arthroplasties are performed every year,^1^ and use of these procedures is anticipated to increase approximately 4-fold by 2030.^2^ To reduce the cost burden of LEJR, the Centers for Medicare & Medicaid Services (CMS) implemented the Comprehensive Care for Joint Replacement (CJR) policy in April 2016, a traditional Medicare bundled payment program that covers an episode of care for a patient who undergoes LEJR. The CJR is the first Medicare bundled payment program that mandated participation of hospitals in randomly selected metropolitan statistical areas (MSAs), providing a novel opportunity to examine the outcomes of the program for patients undergoing LEJR both within and outside traditional Medicare.

Many studies have found that CJR reduced the average cost of an episode of LEJR care in traditional Medicare.^3,4,5,6,7,8^ The savings have been attributed to a decrease in the use of institutional long-term care and an increase in home discharge rates with or without assistance from home health agencies.^3,4,5,6,7,8^ Importantly, studies have found that the program did not compromise the quality of care, with no association with hospital readmission rates, use of ambulatory care, or mortality.^9,10^ As Medicare is the biggest payer for LEJR, the program was also associated with changes in outcomes for patients outside of traditional Medicare.^11,12,13^ The CJR policy has been associated with reduced institutional care and increases in home discharge rates for patients undergoing LEJR with Medicare Advantage or non-Medicare coverage.^11,12,13^

By incentivizing hospitals to standardize an episode of care, the CJR policy was expected to reduce existing racial and ethnic differences in LEJR treatment.^9,14^ For example, Black patients are known to be more likely to be discharged to institutional care, including skilled nursing or inpatient rehabilitation facilities, after surgical treatments compared with their White counterparts.^15,16^ A higher institutional discharge rate among Black patients was found to be associated with their higher readmission rates, suggesting poor discharge planning and care continuity for Black patients.^16^ To date, 2 studies examined whether the CJR policy was associated with different health service use patterns and health outcomes based on patients’ race and ethnicity but only within traditional Medicare.^14,17^ These studies showed that CJR was associated with larger reductions in the use of institutional postdischarge care and readmission rates among Black patients, but not among Hispanic patients, compared with White patients, narrowing existing racial differences in LEJR treatment between Black and White patients.^14,17^ Since these studies were limited to traditional Medicare beneficiaries, they leave open the question of whether CJR was associated with differential changes by patient race and ethnicity outside of the traditional Medicare program.

The present study examined the case of California, where Hispanic persons constitute the largest racial and ethnic group (40.4% of the 2023 population) and have been shown to more likely lack traditional Medicare coverage compared with White persons.^18,19,20^ Historically, Hispanic patients have received less attention in the LEJR literature compared with Black patients, despite being similarly less likely to receive LEJR treatment and experiencing worse outcomes after LEJR compared with non-Hispanic White patients.^21,22^ Although Hispanic patients in California, especially those without traditional Medicare coverage, may have experienced unique changes associated with implementation of the CJR model, no work has assessed this to date. In this study, we examined whether patients undergoing LEJR who are not covered by traditional Medicare in California experienced different inpatient and postacute care after CJR model implementation based on their race and ethnicity, with a specific focus on Hispanic patients.

## Methods

This cohort study’s protocol was approved by the University of Southern California Institutional Review Board and the State of California Committee for the Protection of Human Participants, which waived informed consent based on the infeasibility of obtaining consent and the minimal risk of harm. The findings were reported using the Strengthening the Reporting of Observational Studies in Epidemiology (STROBE) reporting guideline.

### Data Source

We used patient discharge data from the California Department of Health Care Access and Information for all major hip or knee joint replacements (Medicare Severity Diagnosis-Related Group codes 469 and 470) conducted between January 1, 2014, and December 31, 2017. The data contain extensive detail, including treating hospital name and location, dates of admission and discharge, patient age, patient race and ethnicity, primary payer, and discharge status.

### Study Population

The study population included Medicare Advantage and non-Medicare patients admitted to hospitals in California for LEJR between 2014 and 2017 to examine indirect changes associated with CJR. We also verified the direct changes associated with CJR for traditional Medicare patients (Supplement 1). All patients admitted to hospitals in the San Francisco-Oakland-Hayward, Modesto, and Los Angeles-Long Beach-Anaheim MSAs, the 3 MSAs randomly chosen by CMS to participate in the CJR program, were included in the treated group, irrespective of primary payer; those admitted to hospitals in the other 23 MSAs in California were included in the control group. Not all 23 MSAs in the control group were included in the original CJR model randomization; those with low LEJR treatment volume and high Bundled Payment for Care Initiative participation rates were excluded from the randomization. However, as this study is based on data from only 1 state, we included all MSAs not participating in the CJR model in the control group to increase the precision of the study results.^13,23^ Analyses based on the original treated and control MSAs are included in Supplement 1.

Patient race and ethnicity were self-reported and grouped into 6 categories in the dataset: Asian or Pacific Islander; Black; Hispanic; Native American, Eskimo, or Aleut; White; and Other.^24^ We grouped Native American, Eskimo, or Aleut with other because of the small sample sizes. Patients treated at the 37 hospitals participating in the Bundled Payment for Care Initiative were excluded as these hospitals were exempt from CJR. We restricted traditional Medicare and Medicare Advantage patients to beneficiaries aged 65 years or older to reduce bias from unobserved medical conditions that younger patients who qualify for Medicare based on disability might have. For the non-Medicare patient group, we restricted to patients aged 64 years or younger to reduce bias in unobserved health differences among those age-eligible for Medicare but with primary coverage from a commercial payer. Finally, hospitalizations without information on racial and ethnic identity or primary payer were excluded (eFigure 1 in Supplement 1).

### Outcomes

Our primary outcomes of interest were inpatient length of stay and home discharge rates. We used the adjusted length of stay variable in the dataset, which replaced 0 days in the length of stay variable with 1 to give value to patients who were admitted and expected to stay overnight but discharged home on the same day.^24^ To address the skewed distribution of the adjusted length of stay, we used a logarithmic transformation. For home discharge rates, we created a binary indicator of 1 for being discharged home and 0 for otherwise. Home discharge included self-care at home, the use of home health services, and hospice care at home.^24^

### Statistical Analysis

We used event study, difference-in-differences (DID), and triple differences (DDD) approaches to examine relative changes in outcomes among patients from racial and ethnic minority groups compared with non-Hispanic White (hereafter, White) patients before and after the CJR policy implementation within and outside Medicare coverage. Both event study and DID analysis control for time trends in before- and after-policy assessment and intuitively show how the policy contributed to the outcomes of interest in the treated group compared with the control group. The DDD analysis is an extended version of DID and used for estimating differential treatment outcomes for subgroups and assessing the statistical significance of the differences. While the event study shows temporal patterns, the DID and DDD summarize changes in the treatment compared with the control group before and after the policy implementation.

We used the DID and DDD analyses to estimate the differential changes in outcomes associated with the CJR program among patients from racial and ethnic minority groups compared with White patients. Since our primary focus was on Hispanic patients, the subgroup analysis comparing Hispanic and White patients is reported as the main analysis. Other subgroup analyses, including analyses of non-Hispanic Asian patients, non-Hispanic Black patients, and patients of other race and ethnicity, are provided in Supplement 1. All analyses included hospital and quarter-year fixed effects to control for fixed differences in outcomes across hospitals and to flexibly control for general time trends, as well as a set of patient characteristics included in the discharge dataset, age and its square, sex (1 for female and 0 otherwise), race and ethnicity, and whether the patient had a major complication or comorbidity (Medicare Severity Diagnosis-Related Group code 470 or 469) (eAppendix in Supplement 1).

We conducted several sensitivity analyses. First, to test for differential selection of patients from racial and ethnic minority groups across treatment and control hospitals, which may bias our analyses, we conducted event study and DID analysis of changes in the proportion of Hispanic patients and patients from other racial and ethnic minority groups in treatment vs control hospitals after CJR implementation. Prior studies that examined differential associations of the CJR model did not assess changes in the ratio of patients from racial and ethnic minority groups within hospitals before and after policy implementation.^14,17^ If there was a substantial decrease in the proportion of racial and ethnic minority patients, the results could be biased due, for example, to selection of the younger, wealthier, or medically less complex patients. Second, we used a wild cluster bootstrap in the DDD analyses to address potential concerns about inference with a treatment (program participation) that was clustered at the MSA level but with only a few treatment clusters. Other sensitivity analyses included DID and DDD analyses (1) without any age restrictions on the sample, (2) that controlled for admission from an emergency department to control for unobserved health differences, and (3) with an indicator for Medicaid eligibility for non-Medicare patients to control for socioeconomic status that might influence health outcomes.

All statistical results were 2-sided, and results were considered statistically significant at a confidence level of 95%. We used Stata/MP, version 16.1 software (StataCorp LLC) for all analyses. Data were collected and analyzed between October 1 and December 31, 2023.

## Results

The total number of hospitalizations included in the study was 309 834 (mean [SD] age, 68.3 [11.3] years; 39.4% men and 60.6% women; 5.0% Asian or Pacific Islander, 5.1% Black, 14.8% Hispanic, 72.4% White, and 2.8% other race and ethnicity). The number of hospitalizations that occurred in the 3 CJR-participating MSAs was 148 724 (48.0%), and the number of hospitalizations that occurred in the other 23 MSAs was 161 110 (52.0%). Among them, 26.8% were covered by Medicare Advantage, and 37.1% were not covered by Medicare; the remaining 36.1% were covered by traditional Medicare (eTable 1 in Supplement 1). Comparing only Hispanic and White patients, the total number of hospitalizations was 270 245 (mean [SD] age, 68.5 [11.2] years; 40.1% men and 59.9% women), among which, 54.2% were from treated MSAs and 45.8% from control MSAs. Generally, the demographic characteristics of patients in treated and control MSAs did not differ substantially. In both treated and control MSAs, a smaller proportion of Hispanic patients had traditional Medicare coverage (20.5% and 27.2%, respectively) compared with White patients (38.0% and 40.3%, respectively) (Table 1).

**Table 1.** Characteristics of Hispanic and White Patients in Treated and Control MSAs in California, 2014-2017 (N = 270 245)

Characteristic	No. of patients (%)			
	Treatment MSAs (n = 123 778)		Control MSAs (n = 146 467)	
	Hispanic	Non-Hispanic White	Hispanic	Non-Hispanic White
No. of patients	25 039 (16.8)	98 739 (66.4)	20 900 (13.0)	125 567 (77.9)
Age, mean (SD), y	65.7 (11.7)	68.9 (11.2)	65.6 (11.7)	69.2 (10.9)
Sex				
Female	15 680 (62.6)	58 791 (59.5)	12 415 (59.4)	75 089 (59.8)
Male	9359 (37.4)	39 948 (40.5)	8485 (40.6)	50 478 (40.2)
Primary payer				
Traditional Medicare	5132 (20.5)	37 538 (38.0)	5903 (27.2)	50 559 (40.3)
Medicare Advantage	8463 (33.8)	26 574 (26.9)	5354 (25.6)	32 504 (25.9)
Non-Medicare	11 444 (45.7)	34 627 (35.1)	9643 (46.1)	42 504 (33.8)
MS-DRG code				
469, Major joint replacement with MCC	843 (3.4)	3845 (3.9)	686 (3.3)	4724 (3.8)
470, Major joint replacement without MCC	24 196 (96.6)	94 894 (96.1)	20 214 (96.7)	120 843 (96.2)

Abbreviations: MCC, major complication or comorbidity; MSA, metropolitan statistical area; MS-DRG, Medicare Severity Diagnosis-Related Group.

The Figure presents unadjusted quarter-year (3-month) changes in hospital length of stay and home discharge rates of Hispanic and White patients with Medicare Advantage and non-Medicare coverage. These plots are consistent with the parallel trend assumption before the policy implementation and illustrate the potential for different indirect changes associated with the program for Hispanic patients and White patients. Patients in treated MSAs had a longer adjusted length of stay compared with those in control MSAs, regardless of their racial and ethnic identity or primary payer. The gap narrowed after CJR implementation, especially for White patients. A more apparent difference was observed in home discharge rates. Patients in treated MSAs had lower home discharge rates, particularly among Hispanic patients. Before CJR, the gap was larger for Hispanic patients, but it remarkably narrowed after program implementation, suggesting that Hispanic patients outside of traditional Medicare may have experienced more substantial changes associated with CJR (Figure). A similar trend, specifically a notable difference in home discharge rates, was also observed among Hispanic and White patients with traditional Medicare coverage (eFigure 2 in Supplement 1). Event study graphs also showed similar disparities between Hispanic and White patients across different patient groups (eFigures 3 and 4 in Supplement 1).

**Figure.**
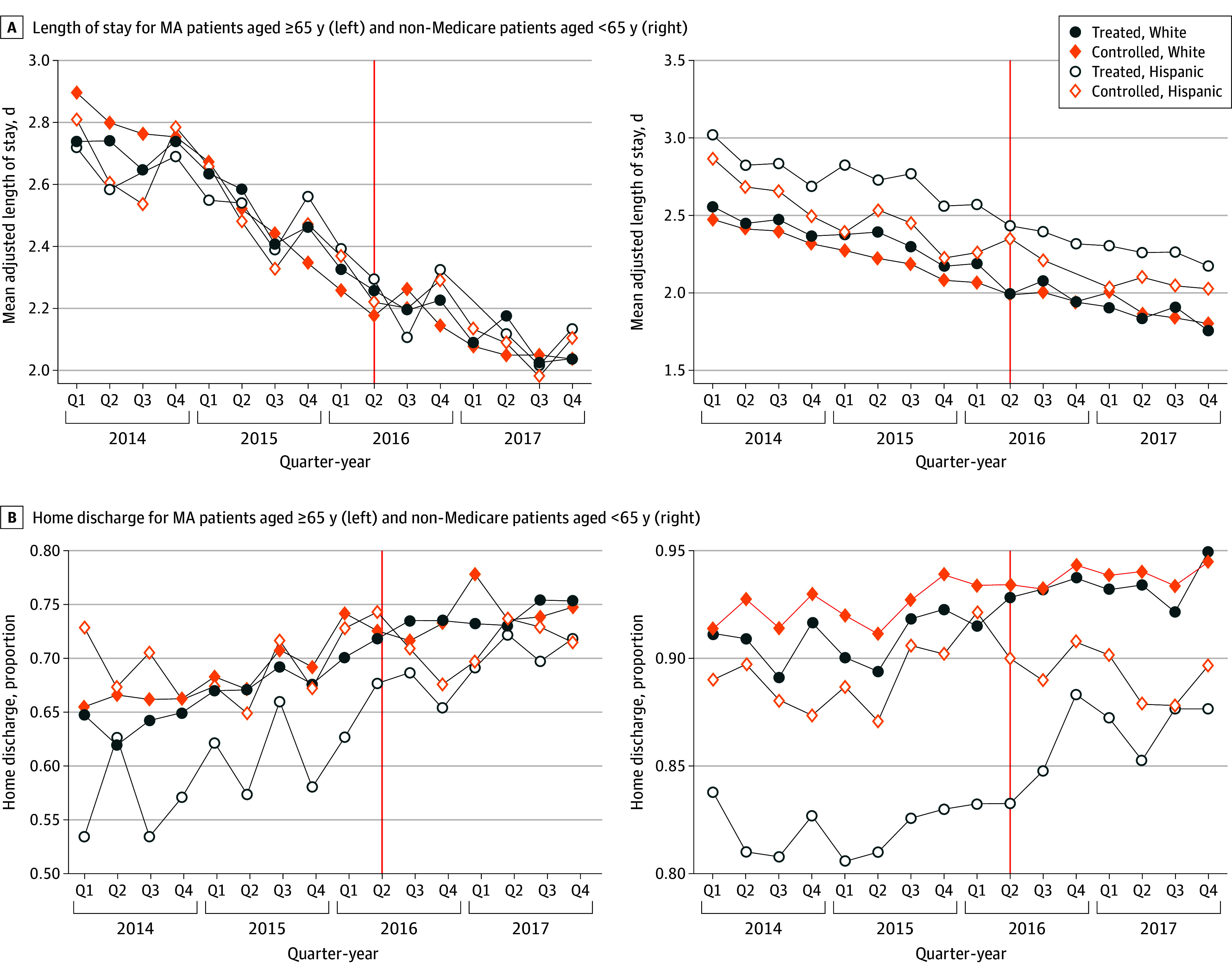
Unadjusted Changes in Health Care Services Use Among Hispanic Patients and White Patients in Treated and Control Metropolitan Statistical Areas (MSAs) in California, 2014-2017 (N = 171 113) The red line indicates the time of the policy (Comprehensive Care for Joint Replacement) implementation, which occurred in the second quarter of 2016. MA indicates Medicare Advantage; Q, quarter.

Table 2 presents DID analyses for Hispanic and White patients with Medicare Advantage and non-Medicare coverage, showing significant differences between the 2 patient groups. For log-adjusted length of stay, the CJR program was associated with a slight, but statistically significant increase of 0.01 (95% CI, 0.00-0.03) days for White patients with Medicare Advantage in treated MSAs compared with control MSAs. Meanwhile, the program was associated with a statistically significant decrease of 0.02 (95% CI, −0.03 to −0.01) days for White patients without Medicare in treated MSAs compared with their counterparts. In contrast, changes among Hispanic patients with Medicare Advantage or non-Medicare coverage were statistically imprecise (Table 2).

**Table 2.** DID Results of Patients With Medicare Advantage and Non-Medicare Coverage in Treated and Control MSAs in California, 2014-2017 (N = 171 113)

Coverage	DID for non-Hispanic White patients				DID for Hispanic patients			
	No. of patients	Coefficient (95% CI)	*P* value	Relative change, %	No. of patients	Coefficient (95% CI)	*P* value	Relative change, %
Log-adjusted length of stay, d								
Medicare Advantage	59 078	0.01 (0.00 to 0.03)	.04	1.0	13 817	0.02 (−0.00 to 0.05)	.12	2.0
Non-Medicare	77 131	−0.02 (−0.03 to −0.01)	.003	−2.0	21 087	−0.01 (−0.03 to −0.01)	.36	−1.0
Home discharge rates, percentage points								
Medicare Advantage	59 078	0.02 (0.01 to 0.03)	.006	2.9	13 817	0.07 (0.04 to 0.10)	<.001	11.9
Non-Medicare	77 131	0.01 (0.00 to 0.02)	.03	1.1	21 087	0.04 (0.02 to 0.05)	<.001	4.9

Abbreviations: DID, difference in differences; MSA, metropolitan statistical area.

For home discharge rates, the CJR program was associated with a relative increase across all patient groups in treated MSAs; however, the increase was larger among Hispanic patients. Higher home discharge rates were found among patients with Medicare Advantage in treated MSAs compared with control MSAs (Hispanic patients, 0.07 [95% CI, 0.04-0.10] percentage points; White patients, 0.02 [95% CI, 0.01-0.03] percentage points). Higher home discharge rates were also observed among patients without Medicare coverage in treated MSAs compared with control MSAs (Hispanic patients, 0.04 [95% CI, 0.02-0.05] percentage points; White patients, 0.01 [95% CI, 0.00-0.02] percentage points) (Table 2). A similar pattern was observed for Hispanic and White patients with traditional Medicare coverage (eTable 2 in Supplement 1).

To test whether the findings from DID analyses were statistically different by race and ethnicity, we used DDD analyses and the wild cluster bootstrap method (Table 3). For the log-adjusted length of stay, no statistically significant difference was found between Hispanic and White patients regarding the relative increase or decrease associated with the CJR implementation. For home discharge rates, however, there was a statistically significant difference between Hispanic and White patients with Medicare Advantage or non-Medicare coverage in treated MSAs regarding the relative increase associated with the program. Hispanic patients with Medicare Advantage and without Medicare had a higher increase of 0.05 (95% CI, 0.02-0.08) percentage points and 0.03 (95% CI, 0.01-0.04) percentage points, respectively, compared with their White counterparts, suggesting that they experienced more substantial changes after the CJR program. The difference in home discharge rates remained statistically significant even after executing 999 replications based on bootstrap samplings with clusters. When executing the same DDD analyses with Hispanic and White patients with traditional Medicare coverage, the results showed no notable difference based on race and ethnicity regarding the changes associated with the CJR policy (eTable 3 in Supplement 1). The DDD analyses with Asian or Pacific Islander and Black patients compared with their White counterparts also showed that only Medicare Advantage– and non-Medicare–covered racial and ethnic minority patients experienced disproportionate changes after the program in their home discharge rates (eTable 4 and 5 in Supplement 1).

**Table 3.** Triple Differences Results of Hispanic and White Patients With Medicare Advantage and Non-Medicare Coverage in Treated and Control MSAs in California, 2014-2017 (N = 171 113)

Coverage	No. of patients	Triple differences		
		Coefficient (95% CI)	*P* value	Wild cluster bootstrap
Log-adjusted length of stay, d				
Medicare Advantage	72 895	0.00 (−0.03 to 0.04)	.80	−0.084 to 0.068
Non-Medicare	98 218	0.01 (−0.01 to 0.04)	.35	−0.037 to 0.047
Home discharge rates, percentage points				
Medicare Advantage	72 895	0.05 (0.02 to 0.08)	.001	0.008 to 0.083
Non-Medicare	98 218	0.03 (0.01 to 0.04)	.002	0.003 to 0.049

Abbreviation: MSA, metropolitan statistical area.

Sensitivity analyses (ie, event study and DID analyses for LEJR receipt) confirmed that there was no substantial change in an access to LEJR treatment among Hispanic patients and patients from other racial and ethnic minority groups across the various insurance coverages (eFigures 5 and 6 and eTables 6 and 7 in Supplement 1). Other sensitivity analyses with larger samples and additional variables generated similar results as the analytic sample (eTables 8-11 in Supplement 1). Finally, sensitivity analyses using CMS’s original CJR treatment and control groups did not show significant differences from our main study results (eTables 12 and 13 in Supplement 1).

## Discussion

The CJR program was expected to standardize care protocols for LEJR procedures in hospitals with the aim of mitigating the prevailing racial and ethnic differences in LEJR treatment. However, the extent to which the CJR policy has realized these expectations remains underexplored. Previous studies focused primarily on traditional Medicare patients and had limited consideration of patient selection in their analyses.^14,17^ Addressing the gap in the existing literature, our cohort study found that Hispanic patients covered outside traditional Medicare had more significant changes associated with the program, experiencing a greater increase in home discharge rates compared with White patients, while access to LEJR treatment among racial and ethnic minority patients in treated relative to control hospitals remained unchanged.

We found no significant reduction in length of stay for Hispanic patients and conflicting results for White patients outside traditional Medicare, which may be due to limited capacity for further reducing length of stay. Specifically, the capitation system, care management, and utilization review used by most Medicare Advantage plans and some commercial insurers may leave limited scope for further reductions. Since changes in length of stay were negligible across all patient groups, the differences between the groups were also not significant.

On the other hand, the larger increase in home discharge rates among Hispanic patients may reflect better social support within their families and communities.^25,26^ However, considering that Hispanic patients historically had significantly lower home discharge rates compared with White patients both in the treated and control MSAs before the CJR model, the increase may simply indicate that Hispanic patients had more room for reduction under the bundled payment system.

Importantly, our findings cannot show whether the differential changes associated with the CJR policy were positive or negative. For example, the larger increase in home discharge rates among Hispanic patients does not necessarily equate to an improvement in well-being or treatment quality. If patients from racial and ethnic minority groups are more likely to need institutional care due to, for example, fewer resources at home, such changes may exacerbate disparities in LEJR quality of care. Further research is needed to examine the welfare outcomes of the disproportionate changes associated with the CJR program.

Our study is novel in several ways. It is the first study to examine the disproportionate changes associated with the CJR program based on patient race and ethnicity outside of traditional Medicare coverage. While a few previous studies examined the association between the CJR program and different outcomes across various racial and ethnic groups,^14,17^ none examined the indirect effects outside traditional Medicare. In contrast, our study showed differential changes among Hispanic compared with White patients with Medicare Advantage and non-Medicare coverage. Our findings suggest that averaging results masks important heterogeneity in changes associated with the program across patient race or ethnicity, both inside and outside traditional Medicare.

Another strength of this study is its emphasis on Hispanic patients, who represent a sizable and growing segment of the US population. Unlike prior studies examining the disproportionate outcomes of the CJR policy for patients from racial and ethnic minority groups, which found that Hispanic patients in traditional Medicare did not have significantly different outcomes compared with White patients,^14,17^ we found that the increase in home discharge rates was larger among Hispanic patients with Medicare Advantage and non-Medicare coverage than among White patients. This finding suggests that drawing conclusions about the disproportional influence of the CJR policy based solely on its intended coverage may be insufficient, particularly when considering Hispanic populations.

Finally, this study was based on complete hospitalization data from California. Thus, the study avoided potential selection bias from sampled data and provided a new perspective on changes in postacute care associated with the CJR program in California, a racially and ethnically diverse state that accounts for more than 10% of Medicare beneficiaries.^27^

### Limitations

Our study has some limitations. First, the random participation of the CJR program was at the MSA level rather than at the individual level, which may have introduced bias to this study due to incomplete randomization. Second, as CMS announced a major change in program participation in February 2018, the study period was limited to the last quarter of 2017. Therefore, we could not test whether the observed disproportionate changes associated with the program persisted for many years. Third, due to limitations in our patient discharge dataset, the study did not include comprehensive information on patients’ underlying health conditions or subsequent outcomes, such as rehospitalizations or death. Fourth, our findings may not be applicable to other regions as the study was only performed in California. Because this study is the first to examine racial and ethnic differences in the indirect outcomes associated with CJR, it may motivate future research based on larger geographic areas.

## Conclusions

This cohort study found that the outcomes associated with the CJR program varied by race and ethnicity for patients outside traditional Medicare. Of note, the program was associated with a larger increase in the home discharge rates of Hispanic patients with Medicare Advantage and non-Medicare coverage compared with their White counterparts. This finding underscores the importance of considering the differential outcomes of Medicare payment policies for vulnerable patient populations beyond the initially targeted groups.

## Notes

Supplement 1. **eFigure 1.** Diagram of Study Sample Selection **eAppendix.** Equations for the Event Study (A), Difference-in-Differences (B) and Triple Differences Analysis (C) **eTable 1.** Characteristics of Patients in Treatment and Control MSAs in California by Race and Ethnicity From 2014 to 2017 **eFigure 2.** Unadjusted Changes in Health Care Services Utilization Among Traditional Medicare Hispanic Patients vs Non-Hispanic White Patients in Treatment and Control MSAs in California From 2014 to 2017 **eFigure 3.** Changes in Health Care Services Utilization Among Non-Hispanic White and Hispanic Medicare Advantage Patients in Treatment Relative to Control MSAs in California From 2014 to 2017 **eFigure 4.** Changes in Health Care Services Utilization Among Non-Hispanic White and Hispanic Non-Medicare Patients in Treatment Relative to Control MSAs in California From 2014 to 2017 **eTable 2.** Difference-in-Differences Analyses Results of Traditional Medicare Patients in Treatment and Control MSAs in California From 2014 to 2017 **eTable 3.** Triple Differences Analyses Results of Traditional Medicare Hispanic vs Non-Hispanic White Patients in Treatment and Control MSAs in California From 2014 to 2017 **eTable 4.** Comparison of the Triple Differences Analyses Results Between Traditional Medicare, Medicare Advantage, and Non-Medicare Non-Hispanic Black vs Non-Hispanic White Patients in Treatment and Control MSAs in California From 2014 to 2017 **eTable 5.** Comparison of the Triple Differences Analyses Results Between Traditional Medicare, Medicare Advantage, and Non-Medicare Non-Hispanic Asian vs Non-Hispanic White Patients in Treatment and Control MSAs in California From 2014 to 2017 **eFigure 5.** Changes in Proportion of Hispanic Patients in Treatment Relative to Control MSAs in California From 2014 to 2017 **eFigure 6.** Changes in Proportion of Racial Minority Patients in Treatment Relative to Control MSAs in California From 2014 to 2017 **eTable 6.** Difference-in-Differences Analyses Results of Change in Proportion of Hispanic Patients in Treatment Relative to Control MSAs in California From 2014 to 2017 **eTable 7.** Difference-in-Differences Analyses Results of Change in Proportion of Racial Minority Patients in Treatment Relative to Control MSAs in California From 2014 to 2017 **eTable 8.** Difference-in-Differences Analyses Results of Traditional Medicare, Medicare Advantage, and Non-Medicare Patients in Treatment and Control MSAs in California From 2014 to 2017 With Full Sample Population **eTable 9.** Comparison of the Triple Differences Analyses Results Between Traditional Medicare, Medicare Advantage, and Non-Medicare Hispanic and Non-Hispanic White Patients in Treatment and Control MSAs in California From 2014 to 2017 With Full Sample Population **eTable 10.** Comparison of the Triple Differences Analyses Results Between Traditional Medicare, Medicare Advantage, and Non-Medicare Hispanic and Non-Hispanic White Patients in Treatment and Control MSAs in California From 2014 to 2017 After Controlling for Admission Source **eTable 11.** Triple Differences Analyses Results Among Non-Medicare Hispanic Patients in Treatment and Control MSAs in California From 2014 to 2017 After Controlling for Admission Source and Medicaid Eligibility **eTable 12.** Difference-in-Differences Analyses Results of Medicare Advantage and Non-Medicare Patients in the Original Treatment and Control MSAs in California From 2014 to 2017 **eTable 13.** Comparison of the Triple Differences Analyses Results Between Medicare Advantage and Non-Medicare Hispanic vs Non-Hispanic White Patients in the Original Treatment and Control MSAs in California From 2014 to 2017

Supplement 2.
Data Sharing Statement
