# Supplementary material for: Outcomes by Race and Ethnicity Following a Medicare Bundled Payment Program for Joint Replacement
Source: JAMA Netw Open. 2024 Sep 17;7(9):e2433962. doi: 10.1001/jamanetworkopen.2024.33962 (PMC11409153; doi:10.1001/jamanetworkopen.2024.33962)
Supplement: Supplement 1. — eFigure 1. Diagram of Study Sample Selection eAppendix. Equations for the Event Study (A), Difference-in-Differences (B) and Triple Differences Analysis (C) eTable 1. Characteristics of Patients in Treatment and Control MSAs in California by Race and Ethnicity From 2014 to 2017 eFigure 2. Unadjusted Changes in Health Care Services Utilization Among Traditional Medicare Hispanic Patients vs Non-Hispanic White Patients in Treatment and Control MSAs in California From 2014 to 2017 eFigure 3. Changes in Health Care Services Utilization Among Non-Hispanic White and Hispanic Medicare Advantage Patients in Treatment Relative to Control MSAs in California From 2014 to 2017 eFigure 4. Changes in Health Care Services Utilization Among Non-Hispanic White and Hispanic Non-Medicare Patients in Treatment Relative to Control MSAs in California From 2014 to 2017 eTable 2. Difference-in-Differences Analyses Results of Traditional Medicare Patients in Treatment and Control MSAs in California From 2014 to 2017 eTable 3. Triple Differences Analyses Results of Traditional Medicare Hispanic vs Non-Hispanic White Patients in Treatment and Control MSAs in California From 2014 to 2017 eTable 4. Comparison of the Triple Differences Analyses Results Between Traditional Medicare, Medicare Advantage, and Non-Medicare Non-Hispanic Black vs Non-Hispanic White Patients in Treatment and Control MSAs in California From 2014 to 2017 eTable 5. Comparison of the Triple Differences Analyses Results Between Traditional Medicare, Medicare Advantage, and Non-Medicare Non-Hispanic Asian vs Non-Hispanic White Patients in Treatment and Control MSAs in California From 2014 to 2017 eFigure 5. Changes in Proportion of Hispanic Patients in Treatment Relative to Control MSAs in California From 2014 to 2017 eFigure 6. Changes in Proportion of Racial Minority Patients in Treatment Relative to Control MSAs in California From 2014 to 2017 eTable 6. Difference-in-Differences Analyses Results of Change in Proportion of [file jamanetwopen-e2433962-s001.pdf]

Supplemental Online Content

Kim N, Jacobson M. Outcomes by race and ethnicity following a Medicare bundled payment program for joint replacement. *JAMA Netw Open*. 2024;7(9):e2433962. doi:10.1001/jamanetworkopen.2024.33962

eFigure 1. Diagram of Study Sample Selection

eAppendix. Equations for the Event Study (A), Difference-in-Differences (B) and Triple Differences Analysis (C)

eTable 1. Characteristics of Patients in Treatment and Control MSAs in California by Race and Ethnicity From 2014 to 2017

eFigure 2. Unadjusted Changes in Health Care Services Utilization Among Traditional Medicare Hispanic Patients vs Non-Hispanic White Patients in Treatment and Control MSAs in California From 2014 to 2017

eFigure 3. Changes in Health Care Services Utilization Among Non-Hispanic White and Hispanic Medicare Advantage Patients in Treatment Relative to Control MSAs in California From 2014 to 2017

eFigure 4. Changes in Health Care Services Utilization Among Non-Hispanic White and Hispanic Non-Medicare Patients in Treatment Relative to Control MSAs in California From 2014 to 2017

eTable 2. Difference-in-Differences Analyses Results of Traditional Medicare Patients in Treatment and Control MSAs in California From 2014 to 2017

eTable 3. Triple Differences Analyses Results of Traditional Medicare Hispanic vs Non-Hispanic White Patients in Treatment and Control MSAs in California From 2014 to 2017

eTable 4. Comparison of the Triple Differences Analyses Results Between Traditional Medicare, Medicare Advantage, and Non-Medicare Non-Hispanic Black vs Non-Hispanic White Patients in Treatment and Control MSAs in California From 2014 to 2017

eTable 5. Comparison of the Triple Differences Analyses Results Between Traditional Medicare, Medicare Advantage, and Non-Medicare Non-Hispanic Asian vs Non-Hispanic White Patients in Treatment and Control MSAs in California From 2014 to 2017

eFigure 5. Changes in Proportion of Hispanic Patients in Treatment Relative to Control MSAs in California From 2014 to 2017

eFigure 6. Changes in Proportion of Racial Minority Patients in Treatment Relative to Control MSAs in California From 2014 to 2017

eTable 6. Difference-in-Differences Analyses Results of Change in Proportion of Hispanic Patients in Treatment Relative to Control MSAs in California From 2014 to 2017

eTable 7. Difference-in-Differences Analyses Results of Change in Proportion of Racial Minority Patients in Treatment Relative to Control MSAs in California From 2014 to 2017

eTable 8. Difference-in-Differences Analyses Results of Traditional Medicare, Medicare Advantage, and Non-Medicare Patients in Treatment and Control MSAs in California From 2014 to 2017 With Full Sample Population

eTable 9. Comparison of the Triple Differences Analyses Results Between Traditional Medicare, Medicare Advantage, and Non-Medicare Hispanic and Non-Hispanic White Patients in Treatment and Control MSAs in California From 2014 to 2017 With Full Sample Population

© 2024 Kim N et al. *JAMA Network Open.*

eTable 10. Comparison of the Triple Differences Analyses Results Between Traditional Medicare, Medicare Advantage, and Non-Medicare Hispanic and Non-Hispanic White Patients in Treatment and Control MSAs in California From 2014 to 2017 After Controlling for Admission Source

eTable 11. Triple Differences Analyses Results Among Non-Medicare Hispanic Patients in Treatment and Control MSAs in California From 2014 to 2017 After Controlling for Admission Source and Medicaid Eligibility

eTable 12. Difference-in-Differences Analyses Results of Medicare Advantage and Non-Medicare Patients in the Original Treatment and Control MSAs in California From 2014 to 2017

eTable 13. Comparison of the Triple Differences Analyses Results Between Medicare Advantage and Non-Medicare Hispanic vs Non-Hispanic White Patients in the Original Treatment and Control MSAs in California From 2014 to 2017

This supplemental material has been provided by the authors to give readers additional information about their work.

© 2024 Kim N et al. *JAMA Network Open.*

eFigure1. **Diagram of Study Sample Selection**

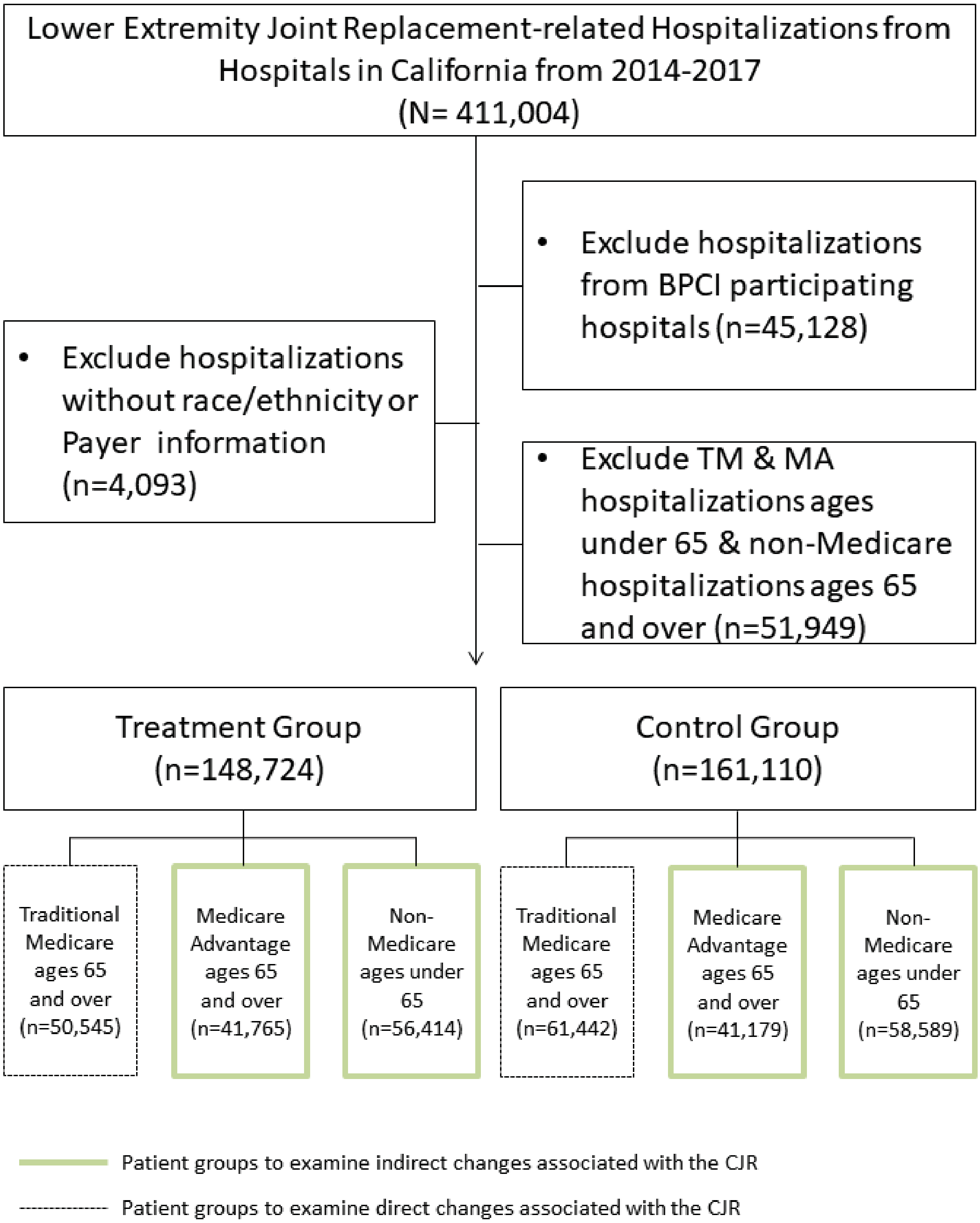

© 2024 Kim N et al. *JAMA Network Open.*

eAppendix. **Equations for the Event Study (A), Difference-in-Differences (B) and Triple Differences Analysis (C)**

Below we show the main regression equations used in this study. In (A), we show the equation for the event study:

$$Y_{ihmq} = a_{hm} + y_q + 8_q * Treated_m + X'_{ihmq}\{3 + E_{ihmq} \quad \text{(A)}$$

where Y_{ihmq} is the outcome of interest (log adjusted length of stay or discharge home) for patient *i* treated in hospital *h* in MSA *m* and quarter-year *q*. The regression includes indicators for hospitals, a_{hm}, to control for fixed differences in outcomes across hospitals and indicators for quarter-year (e.g, Q1 2014; Q2 2014, …) y_q, to flexibly control for general time trends as well as a set of patient characteristics included in the discharge dataset, age and its square, sex (1=female; 0 otherwise), race/ethnicity, and whether the patient had a major complication or comorbidity (MS-DRG 470 or 469). Our interest is in 8_q the coefficients on the quarter-year fixed-effects interacted with the treatment indicator for whether the MSA where the hospital was located was randomized to the CJR treatment. We omitted the interaction term for the first quarter of 2016 such that estimates are normalized to the quarter before CJR took effect. Consequently, the coefficients show the temporal difference in outcomes between treated and control hospitals relative to the reference period and allow us to assess whether our difference-in-differences estimates capture a change in outcomes that is credibly related to CJR.

In (B), we show the equation for the difference-in-differences analysis.

$$Y_{ihmq} = a_{hm} + y_q + oPost_{mq} + X'_{ihmq}\{3 + E_{ihmq} \quad \text{(B)}$$

where Y_{ihmq}, a_{hm}, y_q and X'_{ihmq} are as defined above and $post_{mq}$is an indicator that captures the interaction between the post CJR period (Q2 of 2016 and later) interacted with an indicator for the treated hospitals (i.e., hospitals located in MSAs randomized to CJR). Note that the main post-period effect is subsumed in the quarter indicators and the main MSA effects are subsumed in the hospital indicators. Our key parameter of interest is the coefficient o on $post_{mq}$, which captures the differential change in outcomes in hospitals randomized to CJR after the program was implemented compared to the change for hospitals not randomized to participate in CJR.

In (C), we show the equation for the triple differences analysis:

$$Y_{ihmq} = a_{hm} + y_q + o_{nw} + (Post_{mq} + 1Post_{qnw} + 0Treated_{mnw} +).Post_{mqnw} + X'_{ihmq}\{3 + E_{ihmq} \quad \text{(C)}$$

where Y_{ihmq}, a_{hm}, y_q , X'_{ihmq} and $post_{mq}$ are as defined above. $Post_{qnw}$ is an indicator that captures the interaction between post CJR period (Q2 of 2016 and later) interacted with an indicator for the racial minority patient. $Treated_{mnw}$ is an indicator that captures the interaction between an indicator for the treated hospitals (i.e., hospitals located in MSAs randomized to CJR) interacted with an indicator for the non-White patient. $Post_{mqnw}$ is an indicator that captures the triple interaction between post CJR period and an indicator for the treated hospitals interacted with an indicator for the non-White patient. Our key parameter of interest was is the coefficient $).$ on $post_{mqnw}$, which captures the difference between the

© 2024 Kim N et al. *JAMA Network Open.*

differential change in outcomes among racial minority patients in hospitals randomized to CJR after the program was implemented compared to the change for hospitals not randomized to participate in CJR and the differential change in outcomes among non-Hispanic White patients in hospitals randomized to CJR after the program was implemented compared to the change for hospitals not randomized to participate in CJR.

© 2024 Kim N et al. *JAMA Network Open.*

eTable1. Characteristics of Patients in Treatment and Control MSAs in California by Race and Ethnicity From 2014 to 2017 (N=309,834)

	No. (%)									
	Treated MSAs (n=148,724)					Control MSAs (n=161,110)				
Characteristics	Hispanic	Non-Hispanic Asian/ Pacific Islanders	Non-Hispanic Black	Non-Hispanic White	Others	Hispanic	Non-Hispanic Asian/ Pacific Islanders	Non-Hispanic Black	Non-Hispanic White	Others
No.	25,039 (16.8)	98,739 (66.4)	10,069 (6.8)	10,091 (6.8)	4,786 (3.2)	20,900 (13.0)	125,567 (77.9)	5,305 (3.3)	5,348 (3.3)	3,990 (2.48)
Age, mean (SD)	25,039 (16.8)	10,091 (6.8)	10,069 (6.8)	98,739 (66.4)	67.1 (0.17)	20,900 (13.0)	5,348 (3.3)	5,305 (3.3)	125,567 (77.9)	67.3 (0.18)
Women	65.7 (0.07)	69.9 (0.11)	64.8 (0.11)	68.9 (0.04)	2,956 (61.8)	65.6 (0.08)	67.0 (0.16)	64.0 (0.15)	69.2 (0.03)	2,461 (61.7)
Primary Payer	15,680 (62.6)	6,979 (69.2)	6,522 (64.8)	58,791 (59.5)		12,415 (59.4)	3,747 (70.1)	3,159 (59.5)	75,089 (59.8)	
Traditional Medicare					2,113 (44.2)					1,790 (44.9)
Medicare Advantage	5,132 (20.5)	3,694 (36.6)	2,068 (20.5)	37,538 (38.0)	729 (15.2)	5,903 (27.2)	2,072 (38.7)	1,118 (21.1)	50,559 (40.3)	568 (14.2)
Non-Medicare	8,463 (33.8)	3,130 (31.0)	2,869 (28.5)	26,574 (26.9)	1,944 (40.6)	5,354 (25.6)	1,375 (25.7)	1,378 (26.0)	32,504 (25.9)	1,632 (40.9)
Diagnosis Related Group Code	11,444 (45.7)	3,267 (32.4)	5,132 (51.0)	34,627 (35.1)		9,643 (46.1)	1,901 (35.5)	2,809 (53.0)	42,504 (33.8)	
469, Major joint replacement with MCC					162 (3.4)					163(4.1)
470, Major joint replacement without MCC	843 (3.4)	500 (5.0)	420 (4.2)	3,845 (3.9)	4,624 (96.6)	686 (3.3)	210 (3.9)	167 (3.1)	4,724 (3.8)	3,827 (95.9)

NOTE: MCC = Major Complication or Comorbidity; Others include Native American, Eskimo, Aleut and others.

© 2024 Kim N et al. *JAMA Network Open.*

eFigure 2. Unadjusted Changes in Health Care Services Utilization Among Traditional Medicare Hispanic Patients vs Non-Hispanic White Patients in Treatment and Control MSAs in California From 2014 to 2017 (N = 99,132)

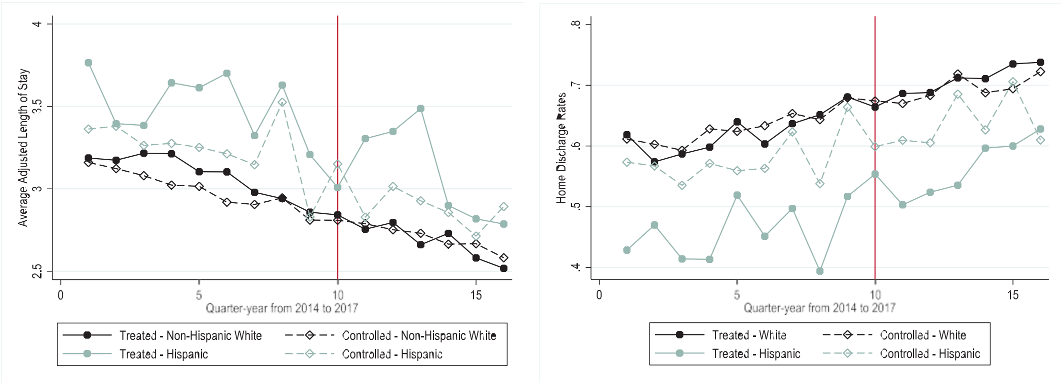

© 2024 Kim N et al. *JAMA Network Open.*

eFigure 3. Changes in Health Care Services Utilization Among Non-Hispanic White and Hispanic Medicare Advantage Patients in Treatment Relative to Control MSAs in California From 2014 to 2017 (N = 72,895)

A) Non-Hispanic White Patients (N=59,078)

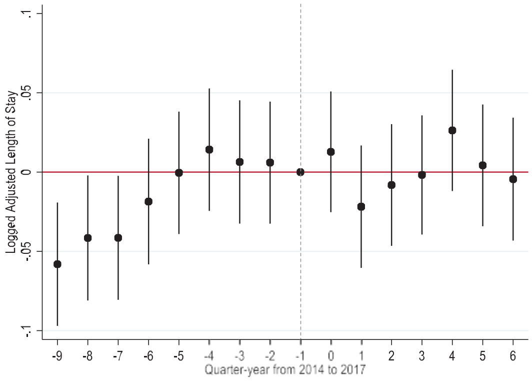

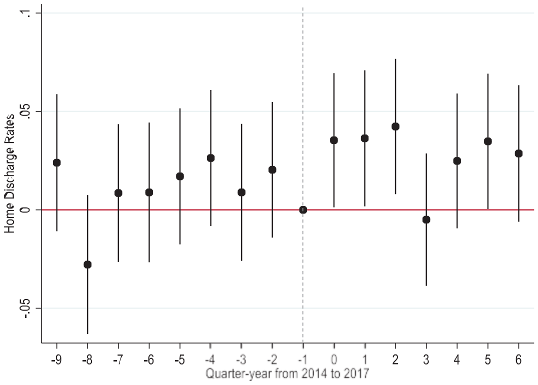

B) Hispanic Patients (N=13,817)

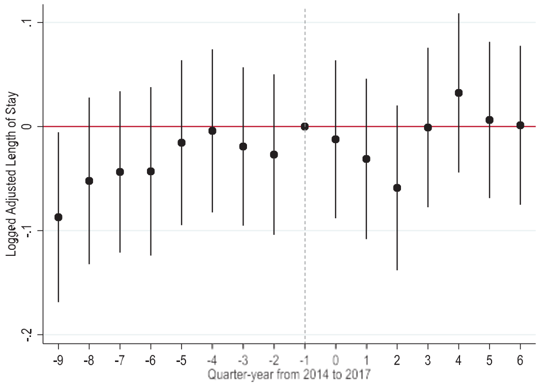

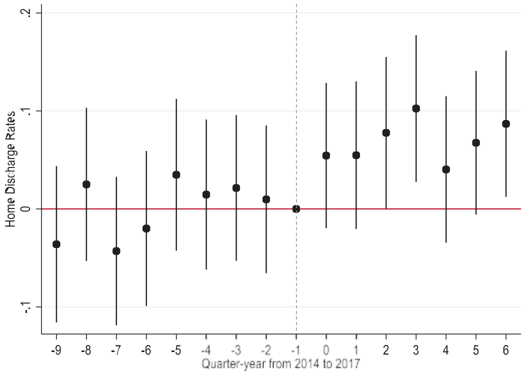

NOTE: 0 quarter-year represents the second quarter of 2016.

© 2024 Kim N et al. *JAMA Network Open.*

eFigure 4. Changes in Health Care Services Utilization Among Non-Hispanic White and Hispanic Non-Medicare Patients in Treatment Relative to Control MSAs in California From 2014 to 2017 (N = 98,218)

A) Non-Hispanic White Patients (N=77,131)

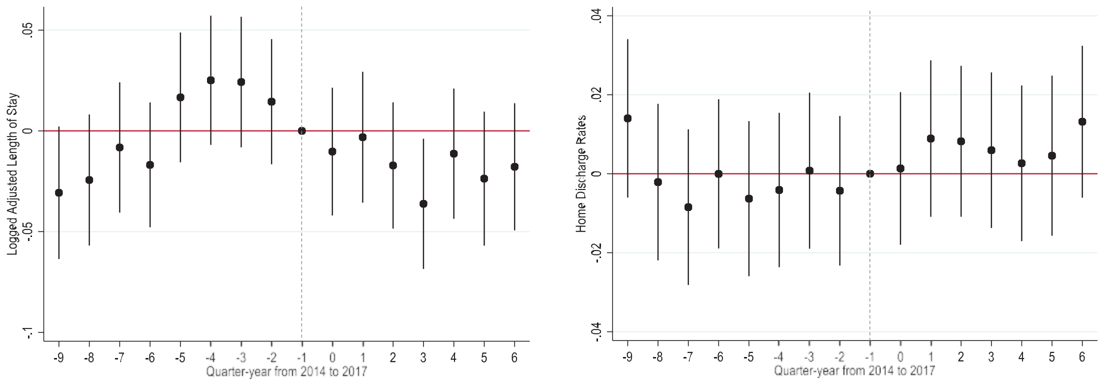

B) Hispanic Patients (N=21,087)

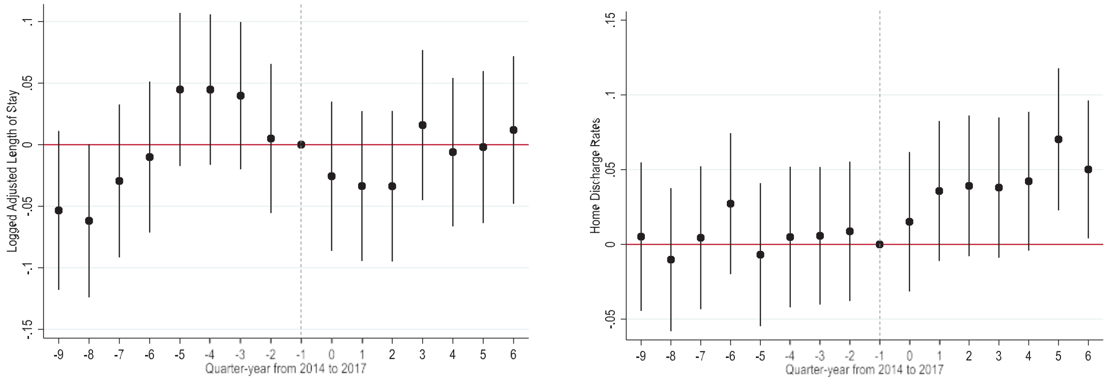

NOTE: 0 quarter-year represents the second quarter of 2016.

© 2024 Kim N et al. *JAMA Network Open.*

eTable 2. Difference-in-Differences Analyses Results of Traditional Medicare Patients in Treatment and Control MSAs in California From 2014 to 2017 (N = 99,132)

	N	Coefficient (95% CI)	*P*	Relative change
Log Adjusted Length of Stay				
DID for non-Hispanic Whites	88,097	-0.04 (-0.05, -0.03)	0.000	4.1%
DID for Hispanics	11,035	-0.03 (-0.06, 0.00)	0.09	3.0%
Home Discharge Rates				
DID for non-Hispanic Whites	88,097	0.03 (0.02, 0.04)	0.000	4.8%
DID for Hispanics	11,035	0.04 (0.00, 0.07)	0.04	8.7%

© 2024 Kim N et al. *JAMA Network Open.*

eTable 3. Triple Differences Analyses Results of Traditional Medicare Hispanic vs Non-Hispanic White Patients in Treatment and Control MSAs in California From 2014 to 2017 (N = 99,132)

	Coefficient (95% CI)	*P*	Wild Cluster Bootstrap
Log Adjusted Length of Stay	0.01 (-0.02, 0.04)	0.50	−0.04, 0.06
Home Discharge Rates	0.01 (-0.02, 0.04)	0.62	−0.04, 0.05

© 2024 Kim N et al. *JAMA Network Open.*

eTable 4. Comparison of the Triple Differences Analyses Results Between Traditional Medicare, Medicare Advantage, and Non-Medicare Non-Hispanic Black vs Non-Hispanic White Patients in Treatment and Control MSAs in California From 2014 to 2017 (N = 239,680)

	N	Coefficient (95% CI)	*P*	Wild Cluster Bootstrap
Log Adjusted Length of Stay				
Traditional Medicare	91,283	0.06 (-0.00, 0.12)	0.05	0.03, 0.10
Medicare Advantage	63,325	-0.03 (-0.09, 0.02)	0.24	−0.11, 0.10
Non-Medicare	85,072	0.03 (-0.01, 0.07)	0.16	−0.04, 0.09
Home Discharge Rates				
Traditional Medicare	91,283	-0.01 (-0.07, 0.05)	0.85	−0.08, 0.06
Medicare Advantage	63,325	0.04 (-0.01, 0.09)	0.10	0.00, 0.09
Non-Medicare	85,072	0.01 (0.00, 0.02)	0.03	−0.04, 0.05

© 2024 Kim N et al. *JAMA Network Open.*

eTable 5. Comparison of the Triple Differences Analyses Results Between Traditional Medicare, Medicare Advantage, and Non-Medicare Non-Hispanic Asian vs Non-Hispanic White Patients in Treatment and Control MSAs in California From 2014 to 2017 (N = 239,745)

	N	Coefficient (95% CI)	*P*	Wild Cluster Bootstrap
Log Adjusted Length of Stay				
Traditional Medicare	93,863	0.03 (-0.01, 0.08)	0.16	−0.04, 0.09
Medicare Advantage	63,583	-0.04 (-0.10, 0.01)	0.12	−0.14, 0.13
Non-Medicare	82,299	0.01 (-0.04, 0.06)	0.70	−0.06, 0.13
Home Discharge Rates				
Traditional Medicare	93,863	0.01 (-0.04, 0.05)	0.72	−0.06, 0.08
Medicare Advantage	63,583	0.06 (0.01, 0.11)	0.03	−0.03, 0.11
Non-Medicare	82,299	0.03 (0.00, 0.06)	0.02	0.02, 0.05

© 2024 Kim N et al. *JAMA Network Open.*

eFigure 5. Changes in Proportion of Hispanic Patients in Treatment Relative to Control MSAs in California From 2014 to 2017 (N = 270,245)

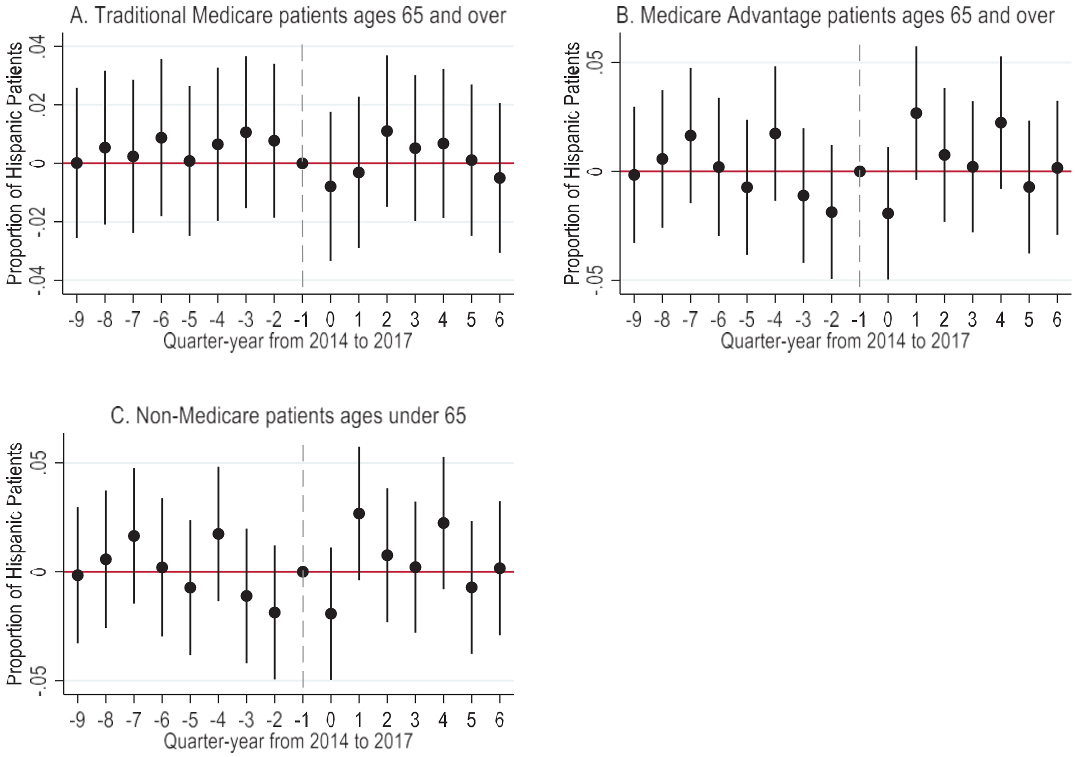

NOTE: 0 quarter-year represents the second quarter of 2016; Analysis was weighted based on the number of admissions per hospital.

© 2024 Kim N et al. *JAMA Network Open.*

eFigure 6. Changes in Proportion of Racial Minority Patients in Treatment Relative to Control MSAs in California From 2014 to 2017 (N = 309,834)

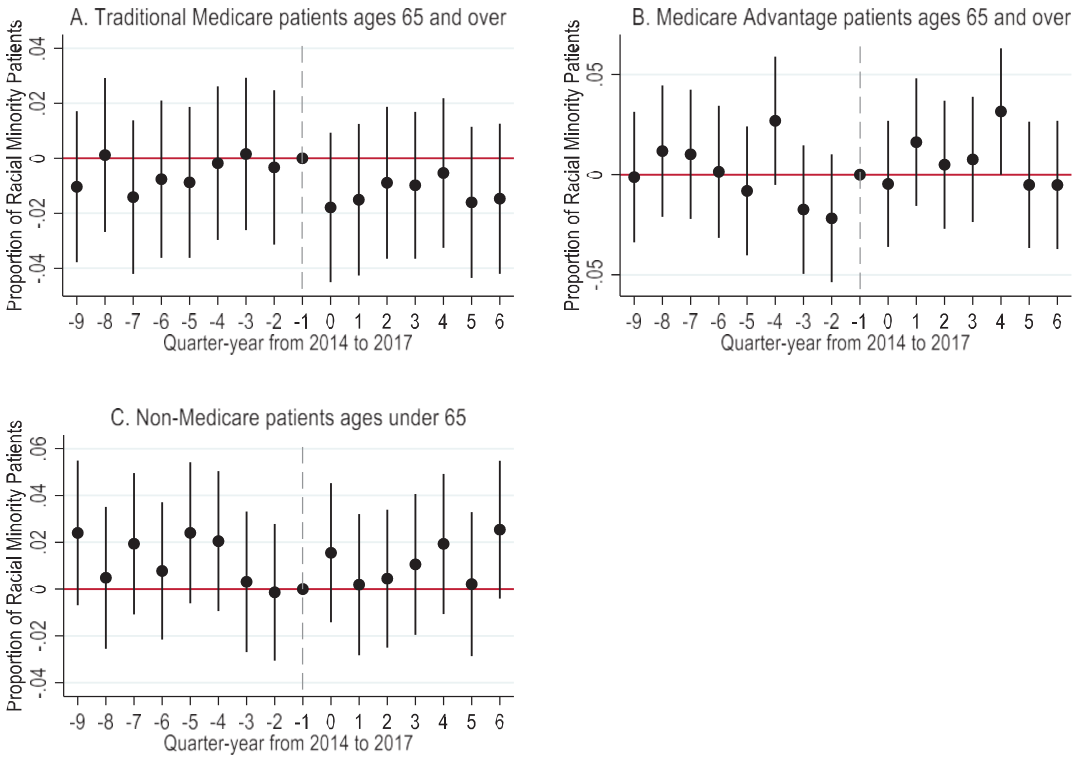

NOTE: 0 quarter-year represents the second quarter of 2016; Analysis was weighted based on the number of admissions per hospital.

© 2024 Kim N et al. *JAMA Network Open.*

eTable 6. Difference-in-Differences Analyses Results of Change in Proportion of Hispanic Patients in Treatment Relative to Control MSAs in California From 2014 to 2017 (N = 270,245)

	N	Coefficient (95% CI)	*P*	Relative Changes
Average Proportion of Hispanic Patients per Hospital				
Traditional Medicare	99,132	-0.003 (-0.01, 0.01)	0.49	-2.5%
Medicare Advantage	72,895	0.005 (-0.01, 0.02)	0.43	2.2%
Non-Medicare	98,218	0.006 (-0.01, 0.01)	0.30	2.5%

NOTE: Analysis was conducted at a hospital-quarter year level; the analysis was weighted based on the number of admissions per hospital.

© 2024 Kim N et al. *JAMA Network Open.*

eTable 7. Difference-in-Differences Analyses Results of Change in Proportion of Racial Minority Patients in Treatment Relative to Control MSAs in California From 2014 to 2017 (N = 309,834)

	N	Coefficient (95% CI)	*P*	Relative Changes
Average Proportion of Racial Minority Patients per Hospital				
Traditional Medicare	111,987	-0.01 (-0.02, 0.00)	0.13	-2.8%
Medicare Advantage	82,944	0.01 (-0.01, 0.02)	0.27	2.6%
Non-Medicare	114,903	0.00 (-0.01, 0.01)	0.90	0.4%

NOTE: Analysis was conducted at a hospital-quarter year level; the analysis was weighted based on the number of admissions per hospital.

© 2024 Kim N et al. *JAMA Network Open.*

eTable 8. Difference-in-Differences Analyses Results of Traditional Medicare, Medicare Advantage, and Non-Medicare Patients in Treatment and Control MSAs in California From 2014 to 2017 With Full Sample Population (N = 306,974)

	DID for non-Hispanic Whites				DID for Hispanics			
	N	Coefficient (95% CI)	*P*	Relative change	N	Coefficient (95% CI)	*P*	Relative change
Log Adjusted Length of Stay								
Traditional Medicare	93,550	-0.04 (-0.05, -0.03)	0.000	-4.1%	13,042	-0.03 (-0.05, 0.00)	0.05	-3.0%
Medicare Advantage	62,027	0.01 (-0.00, 0.03)	0.08	1.0%	15,079	0.08 (0.02, 0.13)	0.01	8.3%
Non-Medicare	96,429	-0.02 (-0.03, -0.01)	0.003	-2.0%	26,847	0.05 (0.00, 0.09)	0.04	5.1%
Home Discharge Rates								
Traditional Medicare	93,550	0.02 (0.01, 0.04)	0.000	3.3%	13,042	0.03 (0.00, 0.07)	0.03	6.4%
Medicare Advantage	62,027	0.02 (0.01, 0.03)	0.002	3.0%	15,079	0.07 (0.01, 0.12)	0.03	13.0%
Non-Medicare	96,429	0.01 (0.01, 0.02)	0.000	1.1%	26,847	0.03 (-0.00, 0.07)	0.07	3.9%

NOTE: Only BPCI participating hospitals were excluded.

© 2024 Kim N et al. *JAMA Network Open.*

eTable 9. Comparison of the Triple Differences Analyses Results Between Traditional Medicare, Medicare Advantage, and Non-Medicare Hispanic and Non-Hispanic White Patients in Treatment and Control MSAs in California From 2014 to 2017 With Full Sample Population (N = 306,974)

	N	Coefficient (95% CI)	*P*	Wild Cluster Bootstrap
Log Adjusted Length of Stay				
Traditional Medicare	106,592	0.01 (-0.02, 0.04)	0.38	−0.05, 0.07
Medicare Advantage	77,106	0.02 (-0.05, 0.09)	0.58	−0.05, 0.10
Non-Medicare	123,276	0.03 (-0.02, 0.07)	0.28	−0.05, 0.10
Home Discharge Rates				
Traditional Medicare	106,592	0.01 (-0.02, 0.04)	0.56	−0.04, 0.05
Medicare Advantage	77,106	0.08 (0.01, 0.14)	0.02	−0.04, 0.14
Non-Medicare	123,276	0.04 (0.01, 0.07)	0.02	0.00, 0.08

© 2024 Kim N et al. *JAMA Network Open.*

eTable 10. Comparison of the Triple Differences Analyses Results Between Traditional Medicare, Medicare Advantage, and Non-Medicare Hispanic and Non-Hispanic White Patients in Treatment and Control MSAs in California From 2014 to 2017 After Controlling for Admission Source (N = 270,245)

	N	Coefficient (95% CI)	*P*	Wild Cluster Bootstrap
Log Adjusted Length of Stay				
Traditional Medicare	99,132	0.02 (-0.02, 0.05)	0.32	−0.05, 0.06
Medicare Advantage	72,895	0.01 (-0.02, 0.04)	0.41	−0.07, 0.07
Non-Medicare	98,218	0.01 (-0.01, 0.04)	0.35	−0.03, 0.05
Home Discharge Rates				
Traditional Medicare	99,132	0.01 (-0.02, 0.04)	0.52	−0.04, 0.05
Medicare Advantage	72,895	0.04 (0.02, 0.07)	0.002	−0.01, 0.08
Non-Medicare	98,218	0.03 (0.01, 0.04)	0.001	0.01, 0.05

© 2024 Kim N et al. *JAMA Network Open.*

eTable 11. Triple Differences Analyses Results Among Non-Medicare Hispanic Patients in Treatment and Control MSAs in California From 2014 to 2017 After Controlling for Admission Source and Medicaid Eligibility (N = 98,218)

	Coefficient (95% CI)	*P*	Wild Cluster Bootstrap
Log Adjusted Length of Stay	0.01 (-0.01, 0.04)	0.28	−0.03, 0.05
Home Discharge Rates	0.02 (0.01, 0.04)	0.002	0.00, 0.04

© 2024 Kim N et al. *JAMA Network Open.*

eTable 12. Difference-in-Differences Analyses Results of Medicare Advantage and Non-Medicare Patients in the Original Treatment and Control MSAs in California From 2014 to 2017 (N = 153,445)

	DID for non-Hispanic Whites				DID for Hispanics			
	N	Coefficient (95% CI)	*P*	Relative change	N	Coefficient (95% CI)	*P*	Relative change
Log Adjusted Length of Stay								
Medicare Advantage	54,725	0.01 (-0.01, 0.02)	0.48	1.0%	13,173	0.01 (-0.02, 0.04)	0.66	1.0%
Non-Medicare	67,101	-0.02 (-0.03, -0.01)	0.004	-2.0%	18,446	-0.01 (-0.04, 001)	0.23	-1.0%
Home Discharge Rates								
Medicare Advantage	54,725	0.02 (0.01, 0.04)	0.001	2.9%	13,173	0.08 (0.05, 0.11)	0.000	12.1%
Non-Medicare	67,101	0.01 (0.00, 0.02)	0.005	1.1%	18,446	0.04 (0.02, 0.05)	0.000	4.3%

© 2024 Kim N et al. *JAMA Network Open.*

eTable 13. Comparison of the Triple Differences Analyses Results Between Medicare Advantage and Non-Medicare Hispanic vs Non-Hispanic White Patients in the Original Treatment and Control MSAs in California From 2014 to 2017 (N=153,445)

		Triple Differences		
		Coefficient (95% CI)	*P*	Wild Cluster Bootstrap
Log Adjusted Length of Stay				
Medicare Advantage	67,898	-0.00 (-0.03, 0.03)	0.92	−0.13, 0.09
Non-Medicare	85,547	0.01 (-0.02, 0.04)	0.50	−0.03, 0.04
Home Discharge Rates				
Medicare Advantage	67,898	0.06 (0.03, 0.09)	0.000	0.01, 0.11
Non-Medicare	85,547	0.02 (0.01, 0.04)	0.007	0.01, 0.04

© 2024 Kim N et al. *JAMA Network Open.*
