# Supplementary material for: Outcomes by Race and Ethnicity Following a Medicare Bundled Payment Program for Joint Replacement
Source: JAMA Netw Open. 2024 Sep 17;7(9):e2433962. doi: 10.1001/jamanetworkopen.2024.33962 (PMC11409153; doi:10.1001/jamanetworkopen.2024.33962)
Supplement: Supplement 2. — Data Sharing Statement [file jamanetwopen-e2433962-s002.pdf]

Data Sharing Statement

Kim. Outcomes by Race and Ethnicity Following a Medicare Bundled Payment Program for Joint Replacement. *JAMA Netw Open*. Published September 17, 2024.
doi:10.1001/jamanetworkopen.2024.33962

Data
Data available: No

Additional Information
Explanation for why data not available: This study analyzed California's Patient Discharge Data (PDD) provided by the California Department of Health Care Access and Information (HCAI) for all hospitalizations for major hip or knee joint replacement (MS-DRG 469 and 470) from 2014 to 2017. To use the dataset, the authors obtained approval from the Committee for the Protection of Human Subjects (CPHS) at the California Health and Human Services.
